# The impact of grandchild caregiving on depression among grandparents: a scoping review

**DOI:** 10.3389/fpubh.2025.1696678

**Published:** 2025-10-31

**Authors:** Jiajing Hu, Nan Zhang, Patreeya Kitcharoen

**Affiliations:** ^1^School of Health Management, Inner Mongolia Medical University, Hohhot, China; ^2^Faculty of Social Sciences and Humanities, Mahidol University, Nakhon Pathom, Thailand

**Keywords:** grandchild caregiving, grandparents, depression, caregiving intensity, caregiving transitions

## Abstract

**Introduction:**

Grandparents' involvement in grandchild caregiving has become an increasingly common social phenomenon worldwide. Compared with non-caregivers, grandparents' depression may be influenced by caregiving responsibilities. However, existing studies have reported inconsistent findings regarding this association.

**Methods:**

Following the PRISMA-ScR guidelines, this scoping review systematically mapped the existing literature on the relationship between grandchild caregiving and grandparents' depression. Eight academic databases were searched, yielding 3,174 records. After four screening stages, 30 eligible studies were included for data extraction, analysis, and synthesis.

**Results:**

The evidence revealed that the effects of grandchild caregiving on grandparents' depression are complex and context-dependent. Differences in caregiving definitions and measurements, grandparent and grandchild characteristics, family structures, and national or cultural contexts contributed to inconsistent results. Six major research gaps were identified: (1) geographical and methodological biases; (2) limited diversity in caregiving measurement; (3) lack of lineage-based analysis (maternal vs. paternal grandparents); (4) insufficient attention to grandchild characteristics; (5) reliance on single depression measures; and (6) inadequate exploration of mediating and moderating mechanisms.

**Discussion:**

Future research should expand geographic coverage, especially in developing regions, and adopt qualitative or mixed-method approaches. It should also diversify caregiving measurements, include lineage-based comparisons, integrate grandchild characteristics, use multidimensional depression tools, and apply theoretical models to explore how caregiving influences mental health. This review is limited by language (English-only publications), exclusion of gray literature, and heterogeneity among included studies, which may reduce comparability.

## 1 Introduction

### 1.1 Background

Globally, grandparents play a important role in family life, particularly in grandchild caregiving, by providing emotional support and practical care (1). When parents do not have time to take care of their children, they often prefer to choose grandparents to take care of their children rather than institutions or non-relatives (2, 3). Traditionally, grandparents played a supportive role in grandchild caregiving, but in contemporary society, an increasing number have taking on primary caregiving responsibilities (4).

Grandchild caregiving is a prevalent phenomenon worldwide, with significant regional variations. In Asian countries, it is a common practice, deeply rooted in cultural and familial traditions (5). In China, where intergenerational caregiving remains a widely accepted norm, over 38% of middle-aged and older adults provide regular care (6). In recent years, the prevalence of grandparental caregiving has also increased in Western societies, including the United States and Europe (7–9). Approximately 2.7 million grandparents in the U.S. serve as primary caregivers for their grandchildren (10), whereas in Europe, less than half of grandparents provide any form of childcare and about a quarter engage in caregiving on a regular basis (11). The involvement of grandparent caregiving is influenced by various micro and macro factors. These include increased female labor force participation (12), improved health and life expectancy of older adults (13), rural–urban migration in developing countries (14), and limited public childcare services and parental leave policies (15–17). Social norms and cultural values also play critical roles. In collectivist societies like China, grandchild caregiving is often viewed as a family duty (18). In individualistic societies, such as the United States and Western Europe, grandchild caregiving is more often considered supplementary (19–21), meaning that it typically serves as additional or supportive childcare rather than a primary responsibility, and is often voluntary or occasional in nature.

Depending on family situations, grandparents take on different roles in grandchild caregiving. The most common classifications are occasional care, co-residential care, and primary caregiving (22–24). Primary caregiving refers to situations where grandparents may assume full-time caregiving or legal guardianship due to the absence of parents caused by migration, divorce, substance abuse, or death (25, 26). Occasional care and co-residential care involve grandparents helping their adult children with caregiving responsibilities, with the main difference being whether they live together. These caregiving roles often differ in the amount of time and effort grandparents invest in childcare, commonly referred to as caregiving intensity (2).

Grandchild caregiving is not only a family responsibility but also has a significant impact on grandparents' mental health (27). This impact is dual in nature, potentially bringing both positive effects and psychological health risks. The direction of these effects depends on factors such as caregiving intensity, social support availability (13), economic and health conditions (28), and adaptability to the caregiving role (29).

On the positive side, caregiving can give grandparents a sense of purpose and enhance self-esteem (18, 30). Many view it as a way to fulfill intergenerational responsibilities and show affection to their families (31). It can also strengthen emotional bonds and reduce loneliness (32). Through interactions with grandchildren, schools, and communities, caregiving helps expand social networks and provides emotional support, which benefits mental wellbeing (33–36).

However, aging reduces physical strength, and caregiving can become physically and psychologically demanding (37). Long-term caregiving is associated with symptoms such as anxiety and depression (38). The time burden, especially for co-residing or primary caregivers, can disrupt daily routines and limit opportunities for social engagement and personal fulfillment (18, 39). Reduced social contact may lead to isolation and greater depression risk (40). Financial stress is also common, particularly in the absence of parental support (41, 42). Differences in parenting philosophies between grandparents and adult children may lead to internal conflicts in balancing the roles of caregiver and elder, which may undermine their authority and create family tension (18, 43, 44). These challenges in role identity can lead to chronic psychological stress. Among all consequences, depression emerges as a particularly serious risk associated with the pressures of grandchild caregiving (13).

While the psychological impact of caregiving varies, depression is one of the most commonly reported and potentially serious outcomes (45). Focusing on depression is essential due to its significant impact on both individuals and society, especially among older adults serving as grandchild caregivers (46). Depression is a common mental health issue that significantly affects quality of life (47) and contributes to the global burden of disease, accounting for the highest proportion of disability-adjusted life years (DALYs) among all mental health conditions (48). The World Health Organization has identified depression as a leading cause of disability worldwide (49–51). Depression is associated with increased morbidity and mortality from chronic diseases such as cardiovascular disease and diabetes (52–54), accelerated cognitive decline and elevated dementia risk (55, 56), higher suicide rates (57), and greater healthcare dependency (58).

Grandchild caregiving has become an important aspect of many grandparents' lives and may have an effect on their depression. In order to provide a more comprehensive understanding of the current state of academic research, identify gaps in the literature, and explore future research directions, this study adopts a scoping review approach (59). The goal is to synthesize and analyze existing literature on the impact of grandchild caregiving on grandparents' depression, thereby contributing to academic understanding and informing empirical research and policy development.

### 1.2 Rationale, aims, and objectives

Preliminary searches across multiple academic databases indicate that, despite the growing prevalence of grandparental caregiving and increasing research interest in its effects on grandparents' physical and mental health, there is currently no scoping review of the effects of grandchild caregiving on grandparents' depression. Existing research has found mixed results on the effects of grandchild caregiving on grandparent depression (60–63). Therefore, conducting a scoping review and synthesis of the existing literature can help clarify the patterns of impact that grandchild caregiving has on grandparents' depression and provide a theoretical foundation for future research in this field.

The aim of this scoping review is to systematically map and synthesize existing research on the relationship between grandchild caregiving and grandparents' depression. Specifically, this review aims to: (1) Identify the geographical distribution, study designs, and methodological approaches of existing studies; (2) Summarize how grandchild caregiving and depression have been defined and measured; (3) Synthesize the main findings regarding the association between caregiving and depression; and (4) Identify key research gaps and propose directions for future inquiry.

Thus, this scoping review is structured around the central question: “What is the impact of grandchild caregiving on depression among grandparents?” By reviewing research methodologies, key findings, and study limitations, this review aims to address gaps in knowledge and offer insights to guide future empirical and policy research.

### 1.3 Scope of the review

This scoping review focuses on the relationship between grandchild caregiving and grandparents' depression. The scope is defined using the Population–Concept–Context (PCC) framework: (a) All grandparents who assume caregiving responsibilities for their grandchildren being the population (P); (b) Grandchild caregiving, including both formal and informal caregiving responsibilities being the concept (C). (c) The depression among grandparents being the context (C). The primary objective of this review is to explore how grandchild caregiving influences the level of depression among grandparents.

## 2 Methods

### 2.1 Information sources

Social science and medical databases were included in the search to ensure comprehensive results. The following databases were included in the scoping review: Wiley Online Library, PubMed, Scopus, Elsevier, Taylor & Francis Online, SAGE Journals Online, Embase, BioMed Central (BMC). The date of the most recent search for all databases was 28 February 2025.

### 2.2 Search strategy

The search strategy of this scoping review was developed based on the focus of studies examining the relationship between grandchild care and grandparent depression. Search terms were carefully selected to ensure comprehensive coverage of relevant studies. The search string used was: [(grandparent^*^ OR grandfather OR grandmother) AND (caregiving OR caregiver^*^ OR grandchild^*^ care raising grandchildren) AND (depression OR depressive symptoms OR mental health]. We focused on studies published between 2015 and 2025. Social phenomena including grandchild care and grandparent depression are affected by factors such as demographic changes, increased life expectancy, and changes in family structure. Limiting the search to the past decade ensured that the research results were based on current empirical evidence and reflected the latest trends and methodological advances.

### 2.3 Eligibility criteria

This scoping review applied specific inclusion and exclusion criteria to ensure the selection of relevant studies addressing the impact of grandchild caregiving on grandparents' depression.

Inclusion criteria: A study was included if it: (1) investigated the relationship between grandchild caregiving and grandparents' depression, (2) provided empirical evidence rather than theoretical discussions or commentaries, (3) used standardized or non-standardized assessment tools to measure depression, (4) did not impose specific age restrictions, as individuals may become grandparents at different life stages (sometimes as early as their 40s). Therefore, all age groups of grandparents were eligible for inclusion, (5) included caregiving grandparents, regardless of whether they were biological or step-grandparents, and (6) was published in a peer-reviewed international journal.

Exclusion criteria: A study was excluded if it: (1) focused solely on individual depressive symptoms (e.g., emotional distress, fatigue, loss of interest) rather than overall depression, (2) primarily examined depression in grandchildren or parents rather than grandparents, (3) did not distinguish grandchild caregiving from general caregiving responsibilities, (4) did not involve a grandparent-grandchild caregiving relationship, (5) was a non-empirical publication (e.g., editorials, opinion pieces, conference abstracts, or letters to the editor), (6) was published in a language other than English, or (7) was not accessible in full text.

### 2.4 Screening and selection process

The screening and selection process consisted of three stages. In the first stage, duplicate articles were excluded. The second stage was based on the titles and abstracts of the articles to ensure the relevance of the studies. Two reviewers (JH and NZ) independently screened titles and abstracts to assess the relevance of each article. Studies were excluded if they did not focus on grandchild caregiving, examined depression in grandchildren or parents, or addressed mental health outcomes unrelated to depression. In the third stage, the full texts of the remaining articles were assessed in detail by the same two reviewers. Any discrepancies in the inclusion decisions were discussed and resolved through consensus with a third reviewer (PK). As this is a scoping review, a formal risk of bias or quality assessment was not conducted, which is consistent with the methodological guidance of PRISMA-ScR. This decision aligns with the objectives of scoping reviews, which are to map the existing evidence and identify research gaps rather than to evaluate the quality of individual studies (59). However, studies with unclear methodology or inappropriate measurement tools were excluded as much as possible during the full-text screening stage.

### 2.5 Data charting and synthesis process

All included studies were read in full to confirm their relevance and to extract key information for analysis. Following the PRISMAScR guidelines, a standardized data extraction framework was developed to ensure consistency across studies. An overview of this framework is presented in Table 1. The extracted information included research focus, methodology, characteristics of participants, geographic location, caregiving context, depression measurement tools, main findings, and potential research gaps. Data extraction was conducted independently by two reviewers (JH and NZ), and any discrepancies were resolved through discussion with a third reviewer (PK) until consensus was reached.

**Table 1:** Overview table guiding data extraction and synthesis in this scoping review.

**No**	**Reference**	**Focus of the research**	**Methodology**	**Characteristics of participants**	**Geographic location of the study**	**Caregiving context**	**Measurement of depression**	**Main findings of the research**	**Potential gap identified**
									

The charted data were synthesized using a narrative and thematic approach guided by the methodological frameworks (64, 65). This process involved iterative reading, comparison, and interpretation of the extracted information to identify recurring themes and patterns across studies. The themes were organized around the main analytical dimensions of grandchild caregiving. This approach was chosen to accommodate the methodological diversity of the included studies and to enable a comprehensive understanding of both consistent and divergent evidence. All synthesized results were carefully reviewed for accuracy and completeness before being summarized in tables and figures presented in Section 3.

## 3 Results

In total, 3,174 potentially relevant records were retrieved. Following the initial search, 894 duplicate entries were removed using reference management software. The remaining 2,280 unique records were subjected to a screening of titles and abstracts to determine their relevance to the topic. During this stage, studies that were clearly unrelated to grandchild caregiving or grandparents' depression were excluded. After this screening process, 86 articles were identified for full-text review.

In the full-text assessment phase, each article was carefully examined to determine whether it met the inclusion criteria. A total of 14 articles were excluded for not focusing on the grandparent population, 22 articles did not address grandchild caregiving, and 4 articles lacked a clear definition or operationalization of grandchild caregiving. Additionally, 16 articles were excluded because they did not investigate depression or depressive symptoms as an outcome.

**Figure 1:**
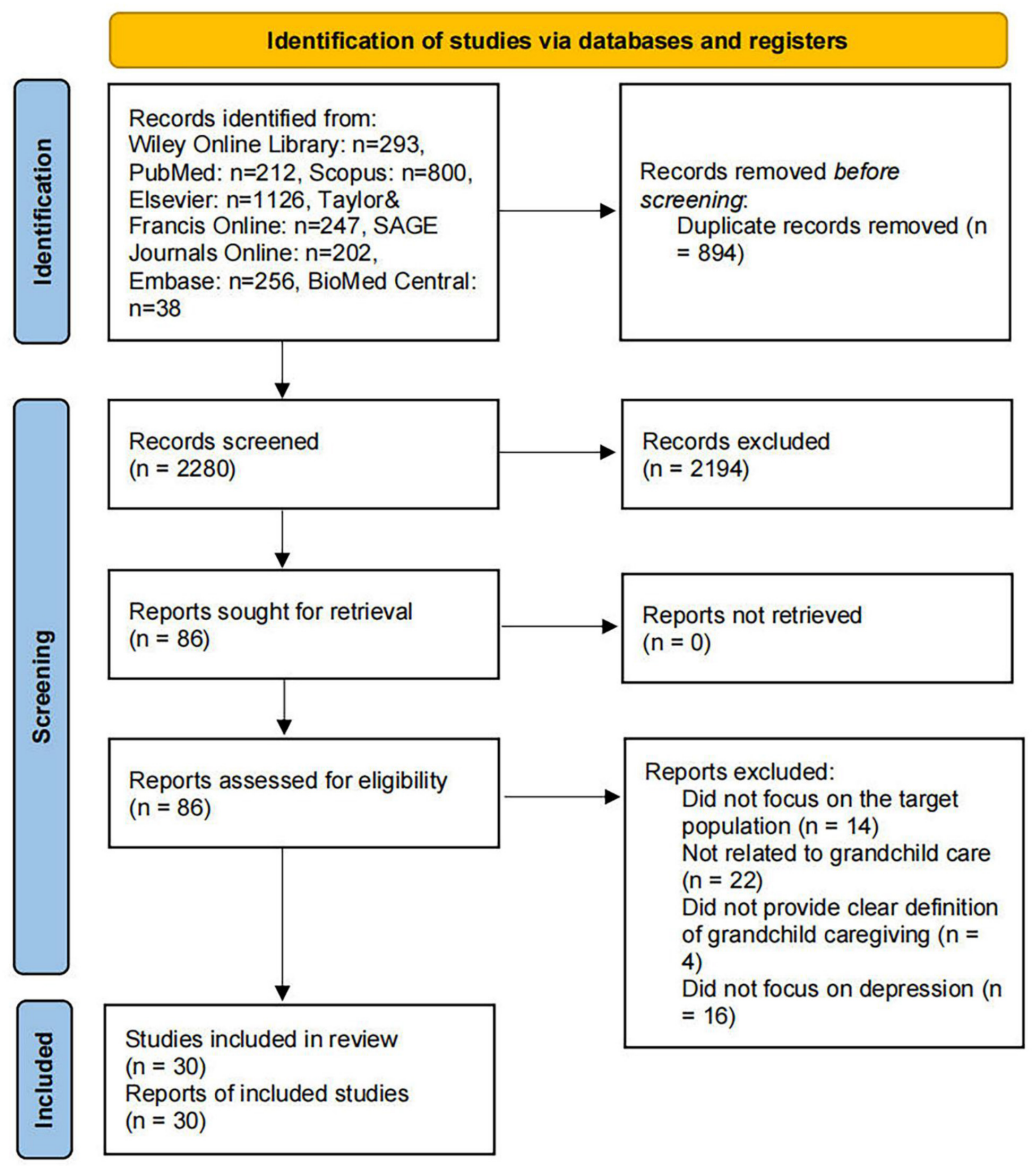
Prisma flow chart.

After applying all exclusion criteria, 30 articles were deemed eligible and were included in the final synthesis for this scoping review. These studies provide a foundation for understanding the existing evidence regarding the impact of grandchild caregiving on depression among grandparents. The flow chart detailing this process is included in Figure 1.

### 3.1 Summary of charted review findings

This section provides an overview of the key features and findings derived from the finalized articles included in this scoping review. Through systematic charting, patterns related to study locations, methodological approaches, participant characteristics, conceptualizations of caregiving, and measurements of depression were synthesized to offer a broad understanding of current research on the relationship between grandchild caregiving and grandparents' depression. The key aggregated characteristics are shown in Table 2, while a detailed summary of each study—including reference, methodology, participants, caregiving definition, depression measurement, and main findings—is provided in the Supplementary Table S1.

**Table 2:** Characteristics of finalized articles.

**Characteristics**	**Frequency (*N*)**	**Percent (%)**
**Geographic location**		
Mainland of China	16	53.33
South Korea	3	10.00
U.S.	4	13.33
Taiwan, China	2	6.67
England	1	3.33
Turkey	1	3.33
European countries (Denmark, Sweden, Austria, France, Germany, Switzerland, Belgium, the Netherlands, Spain, Italy, and Greece)	3	10.00
**Study design**		
Cross-sectional	10	33.33
Longitudinal	20	66.67
**Age of study population**		
No age limit	7	23.33
>45	5	16.67
45–75	1	3.33
45–80	2	6.67
>50	6	20.00
50–74	1	3.33
50–80	2	6.67
50–89	1	3.33
>60	3	10.00
60–80	1	3.33
>65	1	3.33
**Definition of grandchild caregiving**		
Caregiving intensity	24	80.00
Changes in caregiving status	5	16.67
Perceived caregiving burden	2	6.67
**Measurement of depression**		
CES-D	25	83.33
PHQ-9	1	3.33
BDI	1	3.33
EURO-D	3	10.00
**Relation between caregiving and depression**		
Inversely	9	30
Directly	1	6.67
Complicated	18	56.67
Not significant	2	6.67

#### 3.1.1 Geographic location and methods used of the finalized articles

The geographic locations of the finalized studies were determined by the respondents' place of residence and the source of the data. Among the finalized articles, 16 were conducted in mainland China, 14 of which were conducted nationwide (13, 14, 18, 61, 62, 66–74), one based in Beijing (75), and one in Anhui Province (76). Two studies were conducted in Taiwan (77, 78), three in South Korea (79–81), four in the United States (28, 63, 82, 83), one in England (60), and one in Turkey (84). Three studies were conducted across 11 European countries, including Austria, Germany, Sweden, the Netherlands, Spain, Italy, France, Denmark, Switzerland, Belgium, and the Czech Republic (85–87). This pattern indicates that current evidence is largely shaped by research from East Asian contexts, reflecting the strong cultural emphasis on intergenerational caregiving in these societies.

All included studies employed quantitative research designs. Of these, 10 studies used cross-sectional designs (13, 14, 28, 60, 62, 66, 72, 75, 82, 83), while 20 adopted longitudinal designs (18, 61, 63, 67–71, 73, 74, 76–81, 84–87). Most studies relied on secondary data sources (26/30), including large-scale national or regional surveys such as the China Health and Retirement Longitudinal Study (CHARLS) (14, 18, 61, 62, 66–69, 71–74), the Chinese Longitudinal Aging Social Survey (CLASS) (13, 70), the Longitudinal Study of Older Adults in Anhui Province (76), the Health and Retirement Study (HRS) (63), the Korean Longitudinal Study of Aging (KLoSA) (79–81), the Survey of Health, Aging and Retirement in Europe (SHARE) (85–87), the Taiwan Longitudinal Study on Aging (TLSA) (77), the English Longitudinal Study of Aging (ELSA) (60), the National Survey of Families and Households (NSFH) (83), and the Study of Health and Living Status of the Middle-Aged and Elderly in Taiwan (78). The remaining four studies collected primary data through purposive or convenience sampling using face-to-face interviews or questionnaires (28, 75, 82, 84). The widespread use of longitudinal datasets underscores the field's emphasis on tracking caregiving and mental health dynamics over time, although the lack of qualitative or mixed-methods designs limits understanding of subjective caregiving experience.

In term of data analysis, 21 studies reported the statistical software they used. The most commonly used software was Stata (13, 14, 60–63, 67, 69, 73, 74, 76, 86), followed by SPSS (66, 78, 81, 83, 84), Mplus (75, 80, 81), and SAS (79, 82). The analytical methods employed were diverse and included chi-square tests, *t*-tests, ANOVA, regression models (including logistic and linear regression), generalized estimating equation (GEE) analysis, and the Actor–Partner Interdependence Mediation Model (APIMeM). This variety of methods reflects the analytical rigor and methodological diversity across the included studies.

#### 3.1.2 Focus of finalized articles and caregiving context

The finalized articles in this scoping review mainly focused on the impact of grandchild caregiving on grandparents' depression. The finalized articles adopted different definitions and measurement approaches for grandchild caregiving. Overall, the caregiving context was categorized into three main types: caregiving intensity, changes in caregiving status, and perceived caregiving burden. This variation in conceptualization reflects how scholars from different contexts attempted to operationalize the caregiving experience, which in turn shaped the comparability and interpretation of findings across studies.

Twenty-four studies used caregiving intensity as a core indicator (13, 14, 18, 28, 61–63, 66–74, 77, 79, 82–87). Among them, 13 studies defined caregiving as a binary variable—whether grandparents provided care or not (13, 14, 18, 62, 66, 69, 72, 73, 82, 83, 85–87). Three studies measured the frequency of caregiving (76, 77, 85). Seventeen studies assessed caregiving intensity by reporting the number of weeks per year or the average number of hours per week that grandparents spent caring for their grandchildren (13, 14, 28, 61–63, 67–72, 74, 79, 82, 84, 86). In these studies, six treated caregiving time as a continuous variable (13, 14, 62, 69, 72, 82), while 12 studies grouped caregiving intensity based on hours or weeks into categories (13, 61–63, 67, 68, 70, 71, 74, 79, 84, 86). Four studies classified caregiving into high, moderate, and low levels (61, 62, 68, 74), another eight studies used a binary classification to distinguish between intensive and non-intensive caregiving (13, 63, 67, 70, 71, 79, 84, 86). These variations reflect different approaches to measuring caregiving involvement and allow a more detailed understanding of how caregiving intensity relates to depression. These patterns suggest that caregiving intensity was the most common and quantifiable indicator, but the lack of consistency in measurement might contribute to the mixed results observed across studies regarding its association with depression.

Five longitudinal studies investigated changes in caregiving status over time (60, 71, 78, 80, 81). These studies tracked transitions such as starting to provide caregiving, stopping caregiving, continued caregiving, or continued non-caregiving. These patterns were used to analyze how changes in caregiving roles may influence grandparents' depression. Findings from these studies highlight that caregiving transitions can have distinct psychological implications, offering dynamic insights into how shifts in care responsibility affect emotional wellbeing.

Two studies considered perceived caregiving burden, measured through subjective self-reports (75, 82). These included perceived caregiving burden and caregiving stress, which reflect grandparents' emotional experiences beyond objective time measures. Although few in number, these studies underscored the importance of subjective perception, showing that emotional strain rather than caregiving hours may better predict depressive symptoms.

In addition, eight studies examined the roles grandparents played in caregiving (18, 63, 66, 67, 69, 76, 77, 84). Four studies addressed the dual caregiving role, where some grandparents provided care to both their grandchildren and their adult children (i.e., the grandchildren's parents) (18, 66, 69, 77). Another four studies analyzed the role of custodial grandparents based on household structure, where adult children were absent from the household (63, 67, 76, 84). However, the reasons for their absence were not discussed.

Besides examining the direct effects of caregiving on depression, 14 studies included mediating or moderating factors (13, 14, 28, 63, 66, 68, 70, 72, 73, 76, 80, 81, 83, 87), and 8 studies conducted subgroup analyses (18, 61, 62, 67, 71, 74, 79, 84). Mediators included intergenerational support (13, 14, 73), intergenerational emotional comfort, social communication (14), internet use (72), intergenerational contact, social activities (81), grandmother–child conflict (75), and social engagement (63). Moderating factors included residential location (68), financial and emotional support (28, 76), gender, age (68), income (68, 80), marital status (83), and work status (87). Subgroup analyses were conducted based on gender (18, 61, 62, 71, 74), household structure (67, 84), and urban–rural residence (18, 61, 71, 74). Together, these findings indicate that the relationship between caregiving and depression is context-dependent, shaped by both structural conditions and individual perceptions, reinforcing the need for theoretical integration in future research.

#### 3.1.3 Characteristics of participants

Among the 30 finalized articles, 22 articles required participants to have at least one grandchild (13, 18, 28, 60–63, 66, 67, 70–76, 78–81, 83, 86), while the remaining eight studies did not specify whether individuals without grandchildren were excluded (14, 68, 69, 77, 82, 84, 85, 87). One study included only grandparents who provided care for their grandchildren (75). Additionally, two studies required that participants have no functional impairments or difficulties in daily activities (70, 77).

In terms of gender, four studies included only grandmothers as participants (75, 80, 84, 87). The remaining 26 studies included both grandfather and grandmother, with 24 reporting the gender composition of the sample. In most of these studies, the proportion of female participants was higher, while the percentage of male participants typically ranged from 39% to 52%. This gender imbalance reflects the social reality that women are more likely to take on childcare responsibilities, which may also contribute to gendered differences in depressive outcomes.

Regarding age criteria, seven studies did not specify an age limit for participants (13, 18, 62, 63, 75, 83, 87). The other 23 studies defined minimum age thresholds, which varied across studies and included 45 years and older (14, 66, 67, 69, 71, 73, 74, 81), 50 years and older (60, 61, 68, 72, 77–80, 85, 86), 60 years and older (28, 70, 76, 82), and 65 years and older (84). Eight studies also set an upper age limit—five studies used 80 years as the cutoff (67, 68, 70, 72, 81), while the remaining three used 74 (80), 75 (66), and 89 years (85), respectively. Most studies utilized secondary datasets that had predefined age criteria during initial participant recruitment. As a result, age variations across datasets were largely determined by the sampling frameworks of existing surveys, which may constrain the generalizability of findings to younger or very old grandparents.

With respect to the age of grandchildren, only seven studies provided age restrictions. Among them, five studies limited grandchildren's age to under 16 years (18, 61, 62, 73, 76), one to under 15 years (60), and one included grandchildren aged between 7 and 12 years (75). Few studies clearly described grandchildren's developmental stages, which makes it difficult to assess how caregiving demands differ depending on grandchildren's ages.

In terms of research subjects, three studies focused on specific populations, including Chinese Americans (82), Mexican Americans (28), and grandparents in rural areas (76).

#### 3.1.4 Measurement of depression

All of the included studies assessed grandparents' depression using standardized measurement tools. The Center for Epidemiologic Studies Depression Scale (CES-D) was the most frequently used instrument, applied in 25 of the finalized articles. Among these, 3 studies used the original 20-item version (28, 66, 75), 1 studies used a 12-item version (83), 16 studies used the 10-item short form (14, 18, 61, 62, 67–69, 71–74, 77–81), 3 studies used a 9-item version (13, 70, 76), and 2 studies used an 8-item version (60, 63). The frequent use of CES-D highlights its dominance as a standardized measure in this research field, although its variations across studies could affect the comparability of depressive symptom scores.

Among the remaining studies, 3 used the EURO-D scale (85–87), and 1 study each used the Patient Health Questionnaire-9 (PHQ-9) (82) and the Beck Depression Inventory (BDI) (84). In addition, only four studies reported using versions of the measurement tools that were adapted to the local research context (77, 85–87). This limited adaptation suggests that cultural and linguistic factors have not been sufficiently considered in measurement design, which could lead to potential bias in capturing depressive symptoms among diverse populations.

Overall, the dominance of Western-developed scales such as CES-D and BDI, coupled with limited contextual adaptation, indicates a methodological gap.

#### 3.1.5 Main findings in the finalized articles

The findings across the included studies indicate that the relationship between grandchild caregiving and grandparents' depression is complex and varies across different contexts and populations. Only one study found that grandparents who provided care for grandchildren experienced increased depression (75). Specifically, caregiving stress and caregiving intensity were found to elevate depression levels. The evidence reflects a non-linear and context-dependent relationship, suggesting that caregiving can serve both as a source of emotional fulfillment and as a potential stressor depending on the caregiving intensity, family dynamics, and available social support.

Nine studies found that grandchild caregiving was associated with lower level of depression among grandparents (13, 14, 18, 69, 70, 72, 73, 85, 87). This protective effect was more pronounced among certain subgroups, including middle-aged grandparents aged 45–60, those living in rural areas (14), urban grandfathers (18), and non-working grandmother (87). This finding indicates that when caregiving demands exceed coping resources, depressive symptoms are likely to increase.

Two studies reported no significant association between grandchild caregiving and grandparents' depression (76, 86). However, one of these studies found that caregivers reported better self-rated health (86), and the other indicated that caregiving frequency was associated with reduced emotional and cognitive distress in contexts where adult children provided less financial support (76). Such neutral results may indicate that caregiving neither harms nor benefits grandparents' mental health directly, but rather interacts with other contextual factors such as economic responsibility and intergenerational reciprocity.

Eighteen studies presented mixed results (28, 60–63, 66–68, 71, 74, 77–84). While some studies found no significant association in the overall sample, subgroup analyses revealed meaningful differences. Grandchild caregiving was associated with reduced depression among grandparents who lived with a spouse or partner (61), those in skipped-generation households (67), grandparents in multigenerational families where the adult children (i.e., the parents of the grandchildren) were present (84), older grandparents (74), and those who offered limited financial support to their adult children (28). These heterogeneous findings imply that the psychological impact of caregiving depends on household structure, intergenerational financial flows, and the degree of emotional interdependence within families.

The effect of caregiving intensity on depression also varied. Three studies showed that moderate-intensity caregiving was linked to reduced depression (61, 68, 74), while one study found a similar protective effect for high-intensity caregiving (79). This suggests a potential U-shaped relationship, where moderate caregiving provides social engagement benefits, whereas excessive caregiving may trigger emotional strain.

Changes in caregiving status also showed complex associations with depression. Three studies reported that discontinuing grandchild caregiving had a negative effect on grandparents' mental health (60, 78, 80). One study found that initiating or continuing caregiving was associated with reduced depression (81). Another reported that both current and former caregivers had lower levels of depression than those who had never provided care (71). Together, these results indicate that the stability and continuity of caregiving roles may contribute to grandparents' psychological wellbeing, whereas abrupt withdrawal from caregiving might lead to emotional loss or identity disruption.

In addition, several mediating variables were identified. These included grandmother–child conflict (75), social interaction (14, 81), intergenerational support (60, 78, 80), generational contract (81), and Internet use (72), all of which significantly mediated the relationship between grandchild caregiving and grandparents' depression.

### 3.2 Synthesis of the results

Most of the included studies were conducted in China, South Korea, the United States, and various European countries, indicating that existing research has primarily been concentrated in developed regions. Regions such as South America, Eastern Europe, Africa, Oceania, and Asian countries outside East Asia were not represented. The studies covered various aspects of grandchild caregiving, including caregiving intensity, changes in caregiving status, perceived caregiving burden, and caregiving roles. However, relatively few studies focused on caregiving transitions, perceived caregiving burden, or the specific caregiving roles of grandparents.

In most studies, depression was measured using the CES-D. Only a small number of studies used alternative measurement tools or versions adapted to the local context. All included studies used quantitative research methods, with two-thirds adopting longitudinal designs. Most studies were based on nationally representative survey datasets. Stata was the most commonly used software for data analysis.

The findings indicate that the relationship between grandchild caregiving and depression among grandparents is complex. The effects of caregiving may differ across population subgroups and geographic regions and are influenced by various mediating and moderating factors.

## 4 Discussion

The evidence synthesized from the included studies shows a complex relationship between grandchild caregiving and grandparents' depression. The final articles included in this scoping review reported different results regarding the effect of grandchild caregiving on grandparents' depression. These differences may be explained by several key factors. First, there is considerable heterogeneity in the definition and measurement of grandchild caregiving. Some studies focused on caregiving intensity, while others emphasized caregiving transitions or perceived caregiving burden, making direct comparisons difficult. Second, differences in the characteristics of grandparents and grandchildren—such as age, gender, health status, and socioeconomic conditions—may influence the caregiving experience and its impact on mental health. Third, many studies did not fully consider family structure, such as whether the grandparent is a primary caregiver, whether they live with the grandchildren, or whether there is support from other family members. These factors may affect the psychological outcomes of caregiving. Finally, the studies were conducted in different countries and cultural contexts, where social norms, intergenerational expectations, and public support systems vary, all of which may influence the relationship between caregiving and depression.

These inconsistent findings can be better understood through theoretical frameworks that explain the mechanisms by which grandchild caregiving may affect depression. The role strain theory suggests that individuals who take on multiple social roles may experience psychological distress when the demands of these roles conflict or exceed personal resources (88). In the context of caring for grandchildren, grandparents may face competing demands from other roles (such as spouse, employee, or caregiver to an aging parent), which can increase their risk of depression, particularly among grandparents who provide intensive or custodial care. This theory helps interpret findings from studies conducted in individualistic societies, where caregiving is often voluntary and may conflict with personal autonomy and social expectations.

On the other hand, the social engagement theory emphasizes the mental health benefits of participating in meaningful social roles (89). From this perspective, caregiving can bring grandparents a sense of purpose, belonging, and emotional satisfaction, especially when caregiving is culturally valued or supported by others. In collectivist contexts, such as East Asian countries, caregiving is often perceived as a moral duty and an expression of filial piety, which may transform potential role strain into psychological reward. Studies that reported a negative relationship between caregiving and depression often emphasized the emotional bond, intergenerational solidarity, and strengthened social ties that help reduce psychological stress. This cultural dimension highlights how caregiving roles, when socially recognized, can serve as protective factors against loneliness and mental distress.

In summary, these theories suggest that the psychological outcomes of caregiving are not the same for everyone. The interaction between caregiving dimensions (intensity, duration, perceived burden) and cultural values may explain why the same caregiving behavior leads to opposite mental health outcomes across studies. Factors such as willingness to provide care, compatibility of social roles, and access to emotional or instrumental support may mediate or moderate the relationship between caregiving and depression. Therefore, future studies should clearly include theoretical frameworks to guide research design and variable selection, and to better understand when and how caregiving affects grandparents' psychological resilience or vulnerability.

### 4.1 Potential gaps of finalized articles

The primary aim of a scoping review is not to provide a detailed summary of existing findings, but rather to highlight key areas where knowledge is lacking and propose directions for future inquiry (90). In light of the results of this review, this section outlines several areas where further research is needed, along with recommendations to guide subsequent studies.

The first gap identified by this scoping review is related to geographic distribution and research methods. Existing literature mainly focuses on China, South Korea, the United States, and a few European countries, while studies from South America, Africa, Oceania, and other Asian countries beyond East Asia are almost absent. All included studies used quantitative methods, with a lack of qualitative or mixed-methods research. This gap shows that the current evidence base on the relationship between grandchild caregiving and grandparents' depression is geographically biased and methodologically limited. Future research should expand to include underrepresented developing countries or regions. It is also necessary to conduct qualitative research to explore in depth the relationship between grandchild caregiving and depression among grandparents, which may help uncover subjective experiences not easily captured by quantitative methods.

The second gap identified by this scoping review is the limited diversity in how grandchild caregiving is measured. Although most studies used caregiving intensity as an indicator—such as caregiving hours or whether caregiving was provided—there was limited attention to caregiving roles (e.g., whether the grandparent is a legal guardian, whether they provide care for multiple generations) and subjective caregiving burden (e.g., self-perceived stress and exhaustion). No studies explored the reasons why grandparents participated in caregiving. Both objective caregiving input and subjective caregiving experience may influence mental health differently. For instance, voluntary and involuntary caregiving might lead to different depression trajectories. Future studies should develop more diverse classifications and measurements of caregiving, combining factors such as legal caregiving status, family structure, and intergenerational responsibilities. Standardized tools should be used to assess subjective caregiving dimensions, such as stress and burden. More detailed measurements can help identify high-risk groups and provide stronger evidence for psychological interventions and policy development.

The third gap identified by this scoping review is the lack of consideration of grandparental lineage (paternal vs. maternal). None of the included studies differentiated between paternal and maternal grandparents. However, in many cultural and social contexts, paternal and maternal grandparents may differ significantly in caregiving involvement, family expectations, and emotional connections, which may affect their depression risk. Future research should include information on grandparental lineage and examine its influence on caregiving stress, parent–child conflict, access to social support, and depression. By using both quantitative and qualitative methods, researchers can better understand the differences in caregiving experiences between paternal and maternal grandparents and promote culturally sensitive support policies.

The fourth gap identified by this scoping review is the insufficient attention to the characteristics of grandchildren as care recipients. Only a few studies reported the age of grandchildren, but did not include this information in the analytical framework. Most studies did not address key characteristics such as grandchildren's behavior, health, or level of dependency. These factors may have a significant impact on caregiving burden and emotional outcomes. Ignoring these aspects limits a comprehensive understanding of the relationship between caregiving and depression. Future studies should systematically include key characteristics of grandchildren, such as age, behavioral problems, and health status, and treat them as explanatory variables or include them in subgroup analysis. Specifically, studies can be designed to collect dyadic data from both grandparents and grandchildren for more accurate analysis.

The fifth gap identified by this scoping review is the limited approach to depression measurement. Most studies used the CES-D scale. Although a few studies used PHQ-9 or BDI, most treated depression as a single total score without distinguishing between emotional, cognitive, and somatic dimensions. Furthermore, culturally adapted tools were rarely used. Given the cultural sensitivity of depressive symptoms, existing tools may not fully reflect the psychological states of grandparents in different contexts. Future studies should consider using a variety of depression scales and conduct cultural adaptation and validation of these tools. In addition, exploring multiple dimensions of depressive symptoms could provide deeper insights into how caregiving affects mood, thinking patterns, and physical wellbeing.

The sixth gap identified by this scoping review is the insufficient exploration of the mechanisms through which grandchild caregiving affects grandparents' depression. Although more than half of the studies included mediating variables, few examined the role of different types of social support (e.g., emotional vs. instrumental). Only one study analyzed the potential mediating role of digital technology. Future research should develop more systematic theoretical models to explore which variables mediate or moderate the relationship between caregiving stress and depression—especially intergenerational support types, value alignment, and access to digital technology.

### 4.2 Limitations

This scoping review has several limitations. First, only studies published in English were included, which may have led to the exclusion of relevant research published in other languages, particularly from non-English-speaking countries. Second, although eight major academic databases were searched, relevant gray literature or unpublished studies may have been missed, potentially affecting the comprehensiveness of the review. Third, the included studies showed considerable heterogeneity in study design, grandchild care measures, and sample characteristics. This variability limited the comparability of the results and the depth of the analysis.

## 5 Conclusion

This scoping review examined the existing evidence on the impact of grandchild caregiving on depression among grandparents. It identified a growing body of quantitative studies exploring this association across various national contexts, particularly in East Asia, the United States, and parts of Europe. The findings suggest that grandchild caregiving has complex and context-dependent effects on grandparents' depression, with both positive and negative associations reported.

Importantly, several notable research gaps were identified, including limited geographic diversity, an overreliance on quantitative methods, insufficient attention to caregiving role types and subjective burden, a lack of lineage-based analyses distinguishing maternal and paternal grandparents, and insufficient exploration of the mechanisms of influence. Addressing these limitations requires methodologically diverse research that integrates both quantitative and qualitative evidence to capture the emotional, relational, and cultural dimensions of caregiving.

From a policy and practice perspective, the findings highlight grandparents as essential contributors to grandchildren's care. Measures such as reducing caregiving burdens, strengthening intergenerational support networks, and expanding community engagement for older caregivers should be used to improve grandparents' mental health.

Future research should expand to underrepresented regions, adopt mixed or qualitative methods, and explore the mechanisms through which caregiving impacts depression. It is also essential to consider the characteristics of grandchildren, the multidimensional nature of depression, and the potential moderating role of social support and digital technology. By addressing these gaps, future studies can contribute to a more comprehensive understanding of how caregiving responsibilities shape the mental health of aging populations.

## Data Availability

The original contributions presented in the study are included in the article/Supplementary material, further inquiries can be directed to the corresponding authors.

## References

[1] Hayslip B Jr Fruhauf CA Dolbin-MacNab ML. Grandparents raising grandchildren: what have we learned over the past decade? Gerontologist. (2017) 59:e152–e63. 10.1093/geront/gnx106 28666363

[2] Chan ACY Lee S-K Zhang J Banegas J Marsalis S Gewirtz AH. Intensity of grandparent caregiving, health, and well-being in cultural context: a systematic review. Gerontologist. (2022) 63:851–73. 10.1093/geront/gnac026 35176155

[3] Likoko S Akokuwebe ME Osuafor GN Idemudia ES. “Health outcomes of grandparents caring for double orphans in South Africa”: what are the determinants? Int J Environ Res Public Health. (2023) 20:7158. 10.3390/ijerph20247158 38131710 PMC10743013

[4] Crowther MR Ford CD Peterson T. A qualitative examination of barriers for urban and rural custodial grandparents. J Intergener Relatsh. (2014) 12:207–26. 10.1080/15350770.2014.929938 26401123 PMC4577064

[5] Chen F Liu G. The health implications of grandparents caring for grandchildren in China. J Gerontol. (2011) 67B:99–112. 10.1093/geronb/gbr132 22156630 PMC3267025

[6] Duan H. The shape of care: patterns of family caregiving among Chinese adults in the middle to later stage of life. Innov Aging. (2021) 5:794–5. 10.1093/geroni/igab046.2930

[7] Mitchell W. The role played by grandparents in family support and learning: considerations for mainstream and special schools. Supp Learn. (2008) 23:126–35. 10.1111/j.1467-9604.2008.00383.x

[8] Bureau USC. Acs 1-Year Estimates Detailed Tables Grandchildren under 18 Years Living with a Grandparent Householder by Age of Grandchild. American Community Survey (2021). Available online at: https://data.census.gov/table/ACSDT1Y2021.B10001?q=ACSDT1Y2018.B10001&g=010XX00US (Accessed September 1, 2025).

[9] Hank K Buber I. Grandparents caring for their grandchildren: findings from the 2004 survey of health, ageing, and retirement in Europe. J Fam Issues. (2009) 30:53–73. 10.1177/0192513X08322627

[10] Ellis RR Simmons T. Coresident Grandparents and Their Grandchildren: 2012 (2014).

[11] Zanasi F Arpino B Bordone V Hank K. The prevalence of grandparental childcare in Europe: a research update. Eur J Ageing. (2023) 20:37. 10.1007/s10433-023-00785-8 37749271 PMC10519902

[12] Shangguan Y Han C Zhao J. Grandparents' retirement and mother's employment quality: the mediating role of intergenerational caregiving. J Fam Issues. (2024) 45:2100–30. 10.1177/0192513X231195654

[13] Wang H Lu Y Huang J. All in the family: does grandparenting impact Chinese grandparents' depressive symptoms? SAGE Open. (2024) 14:21582440241242561. 10.1177/21582440241242561

[14] Wang L Tang Y. Impacts of intergenerational caregiving on grandparents' health: implications for Sdg-3. Econ Anal Policy. (2023) 79:584–98. 10.1016/j.eap.2023.06.015

[15] Jappens M Van Bavel J. Regional family norms and child care by grandparents in Europe. Demogr Res. (2012) 27:85–120. 10.4054/DemRes.2012.27.4

[16] Schwarz B Trommsdorff G Zheng G Shi S. Reciprocity in intergenerational support: a comparison of Chinese and German adult daughters. J Fam Issues. (2010) 31:234–56. 10.1177/0192513X09347991

[17] Zanasi F Arpino B Pirani E Bordone V. Work histories and provision of grandparental childcare among italian older women. Genus. (2022) 78:11. 10.1186/s41118-022-00158-6

[18] Xu H. Physical and mental health of Chinese grandparents caring for grandchildren and great-grandparents. Soc Sci Med. (2019) 229:106–16. 10.1016/j.socscimed.2018.05.047 29866373 PMC6261790

[19] Jendrek MP. Grandparents who parent their grandchildren: effects on lifestyle. J Marr Fam. (1993):609–21. 10.2307/353342

[20] Pruchno R. Raising grandchildren: the experiences of black and white grandmothers. Gerontologist. (1999) 39:209–21. 10.1093/geront/39.2.209 10224717

[21] Xie D Wang J. The association between grandchild care and biological aging among middle-aged and older adults in China. Innov Aging. (2024) 8:igae059. 10.1093/geroni/igae059 39036790 PMC11258899

[22] Noriega C Velasco C Pérez-Rojo G López J. Character strengths and social support as protective factors between grandparents' caregiving and health-related quality of life. J Child Fam Stud. (2022) 31:2505–17. 10.1007/s10826-021-02187-9

[23] Floridi G. Daily grandchild care and grandparents' employment: a comparison of four European child-care policy regimes. Ageing Soc. (2022) 42:448–79. 10.1017/S0144686X20000987

[24] Bell L Mortimer S Matwiejczyk L Moores CJ Prichard I Mehta K . Primary caregiving grandmothers' perspectives of grandchildren's healthy lifestyle behaviours: a qualitative study. J Fam Stud. (2022) 28:19–36. 10.1080/13229400.2019.1673795

[25] Smith GC Hayslip B Jr Webster BA. Psychological difficulties among custodial grandchildren. Child Youth Serv Rev. (2019) 104:104390. 10.1016/j.childyouth.2019.104390 32489225 PMC7265777

[26] Sneed RS Schulz R. Grandparent caregiving, race, and cognitive functioning in a population-based sample of older adults. J Aging Health. (2019) 31:415–38. 10.1177/0898264317733362 29254404 PMC6474833

[27] García-Sanjuán S Gutiérrez-García AI Cabañero-Martínez MJ Fernández-Alcántara M. Rocamora-Rodriguez MdC, Escribano S. Lived Experience of being a grandparent in one region of spain: a qualitative study. Front Public Health. (2024) 12:1419207. 10.3389/fpubh.2024.1419207 39664559 PMC11632530

[28] Liu Y Cong Z. Grandparenting and depressive symptoms among Mexican American older adults: examining the moderating effects of financial and emotional support. J Intergener Relatsh. (2019) 17:163–77. 10.1080/15350770.2018.1535353

[29] Baker LA Silverstein M. Preventive health behaviors among grandmothers raising grandchildren. J Gerontol B Psychol Sci Soc Sci. (2008) 63:S304–11. 10.1093/geronb/63.5.S304 18818451 PMC2633920

[30] Xie J Liao J Zhang J Gu J. Association between rural-to-urban migration and the cognitive aging trajectories of older Chinese adults: results from a prospective cohort analysis. BMC Geriatr. (2020) 20:360. 10.1186/s12877-020-01772-9 32957920 PMC7507287

[31] Duflos M Giraudeau C. Using the intergenerational solidarity framework to understand the grandparent-grandchild relationship: a scoping review. Eur J Ageing. (2022) 19:233–62. 10.1007/s10433-021-00626-6 35663914 PMC9156599

[32] Quirke E König HH Hajek A. Extending understanding of grandchild care on feelings of loneliness and isolation in later life: a literature review. Z Gerontol Geriatr. (2021) 54:513–6. 10.1007/s00391-020-01776-5 32856121 PMC7451229

[33] Wang G Duan J Kan Q Zhou Y Cheng Z Tang S. The correlation analysis of wechat usage and depression among the middle-aged and elderly in China: the mediating role of social participation. BMC Public Health. (2023) 23:462. 10.1186/s12889-023-15349-9 36899336 PMC9999613

[34] Guo J Yang Y Lin L Zhang Y Shen T. Prevalence and factors influencing disability and cognitive impairment among empty nesters and non-empty nesters in Guangdong, China: a cross-sectional study. Front Public Health. (2025) 13:1545497. 10.3389/fpubh.2025.1545497 40213426 PMC11983516

[35] Roberto KA Stroes J. Grandchildren and grandparents: roles, influences, and relationships. Int J Aging Hum Dev. (1992) 34:227–39. 10.2190/8cw7-91wf-e5qc-5ufn 1582715

[36] Xu L Chi I. Determinants of support exchange between grandparents and grandchildren in rural China: the roles of grandparent caregiving, patrilineal heritage, and emotional bonds. J Fam Issues. (2018) 39:579–601. 10.1177/0192513X16662102

[37] Liu Z Heffernan C Tan J. Caregiver burden: a concept analysis. Int J Nurs Sci. (2020) 7:438–45. 10.1016/j.ijnss.2020.07.012 33195757 PMC7644552

[38] Taylor C Kong AC Foster J Badawi N Novak I. Caregivers' feeding experiences and support of their child with cerebral palsy. J Child Fam Stud. (2022) 31:819–30. 10.1007/s10826-021-02123-x 34629833 PMC8489792

[39] Song IH Kang KA. Research trends over 10 years (2010-2021) in infant and toddler rearing behavior by family caregivers in South Korea: text network and topic modeling. Child Health Nurs Res. (2023) 29:182–94. 10.4094/chnr.2023.29.3.182 37554086 PMC10415838

[40] Schulz R Eden J National Academies of Sciences E Medicine. Family Caregiving Roles and Impacts. Families Caring for an Aging America. Washington, DS: National Academies Press (2016). 27905704

[41] Butler FR Zakari N. Grandparents parenting grandchildren: assessing health status, parental stress, and social supports. J Gerontol Nurs. (2005) 31:43–54. 10.3928/0098-9134-20050301-09 15799636

[42] Xu Y Wu Q Levkoff SE Jedwab M. Material hardship and parenting stress among grandparent kinship providers during the Covid-19 pandemic: the mediating role of grandparents' mental health. Child Abuse Negl. (2020) 110:104700. 10.1016/j.chiabu.2020.104700 32854948 PMC7444952

[43] Hayslip B Jr Rodriguez JM Fassi J. Parental style, grandchild problematic behaviors, and parental role demands among grandparent caregivers. Int J Aging Hum Dev. (2025) 28:914150241313312. 10.1177/00914150241313312 39871701

[44] Pearce LD Hayward GM Chassin L Curran PJ. The increasing diversity and complexity of family structures for adolescents. J Res Adolesc. (2018) 28:591–608. 10.1111/jora.12391 30197489 PMC6124501

[45] Cuijpers P Stringaris A Wolpert M. Treatment outcomes for depression: challenges and opportunities. Lancet Psychiatry. (2020) 7:925–7. 10.1016/S2215-0366(20)30036-5 32078823

[46] Herrman H Patel V Kieling C Berk M Buchweitz C Cuijpers P . Time for united action on depression: a lancet-world psychiatric association commission. Lancet. (2022) 399:957–1022. 10.1016/S0140-6736(21)02141-3 35180424

[47] Lépine J-P Briley M. The increasing burden of depression. Neuropsychiatr Dis Treat. (2011) 7:3–7. 10.2147/NDT.S19617 21750622 PMC3131101

[48] GBD. Global, regional, and national burden of 12 mental disorders in 204 countries and territories, 1990–2019: a systematic analysis for the global burden of disease study 2019. Lancet Psychiatry. (2022) 9:137–50. 10.1016/S2215-0366(21)00395-3 35026139 PMC8776563

[49] Seligman F Nemeroff CB. The interface of depression and cardiovascular disease: therapeutic implications. Ann N Y Acad Sci. (2015) 1345:25–35. 10.1111/nyas.12738 25809518

[50] Smith K. Mental health: a world of depression. Nature. (2014) 515:180. 10.1038/515180a 25391942

[51] Astafeva D Kolsanov A Chaplygin S Yashikhina A Cumming P Vlasov A . The efficacy of mobile phone-based interventions for the treatment of depression: a systematic meta-review of meta-analyses of randomized controlled trials. Psychiatr Danub. (2022) 34:155–63. 36170722

[52] Cai J Zhang S Wu R Huang J. Association between depression and diabetes mellitus and the impact of their comorbidity on mortality: evidence from a nationally representative study. J Affect Disord. (2024) 354:11–8. 10.1016/j.jad.2024.03.003 38447915

[53] Qiu W Cai A Li L Feng Y. Association of depression trajectories and subsequent hypertension and cardiovascular disease: findings from the charls cohort. J Hypertens. (2024) 42:432–40. 10.1097/HJH.0000000000003609 37937504

[54] Senoo K Kaneko H Ueno K Suzuki Y Okada A Fujiu K . Sex differences in the association between depression and incident cardiovascular disease. JACC: Asia. (2024) 4:279–88. 10.1016/j.jacasi.2023.11.015 38660110 PMC11035952

[55] Chen C Tian Y Ni L Xu Q Hu Y Peng B. The influence of social participation and depressive symptoms on cognition among middle-aged and older adults. Heliyon. (2024) 10:e24110. 10.1016/j.heliyon.2024.e24110 38293386 PMC10825423

[56] Yin J John A Cadar D. Bidirectional associations of depressive symptoms and cognitive function over time. JAMA Network Open. (2024) 7:e2416305–e. 10.1001/jamanetworkopen.2024.16305 38861255 PMC11167501

[57] Louis F Rongqin Y Abdo U Michael S Seena F. Risk factors for suicide in adults: systematic review and meta-analysis of psychological autopsy studies. Evid Based Ment Health. (2022) 25:148–55. 10.1136/ebmental-2022-300549 36162975 PMC9685708

[58] Jain S Gupta S Li VW Suthoff E Arnaud A. Humanistic and economic burden associated with depression in the United States: a cross-sectional survey analysis. BMC Psychiatry. (2022) 22:542. 10.1186/s12888-022-04165-x 35953786 PMC9367058

[59] Tricco AC Lillie E Zarin W O'Brien KK Colquhoun H Levac D . Prisma extension for scoping reviews (Prisma-Scr): checklist and explanation. Ann Intern Med. (2018) 169:467–73. 10.7326/M18-0850 30178033

[60] Di Gessa G Bordone V Arpino B. Changes in grandparental childcare during the pandemic and mental health: evidence from England. J Gerontol B Psychol Sci Soc Sci. (2023) 78:319–29. 10.1093/geronb/gbac104 36124835 PMC9494312

[61] Tang F Li K Jang H Rauktis MB. Depressive symptoms in the context of Chinese grandparents caring for grandchildren. Aging Ment Health. (2022) 26:1120–6. 10.1080/13607863.2021.1910788 33843385

[62] Zhao D Zhou Z Shen C Ibrahim S Zhao Y Cao D . Gender differences in depressive symptoms of rural Chinese grandparents caring for grandchildren. BMC Public Health. (2021) 21:1838. 10.1186/s12889-021-11886-3 34635088 PMC8507248

[63] Notter IR. Grandchild care and well-being: gender differences in mental health effects of caregiving grandparents. J Gerontol B Psychol Sci Soc Sci. (2022) 77:1294–304. 10.1093/geronb/gbab164 34508596 PMC9255931

[64] Levac D Colquhoun H O'Brien KK. Scoping studies: advancing the methodology. Implement. Sci. (2010) 5:69. 10.1186/1748-5908-5-69 20854677 PMC2954944

[65] Arksey H O'Malley L. Scoping studies: towards a methodological framework. Int J Soc Res Methodol. (2005) 8:19–32. 10.1080/1364557032000119616

[66] Zhao H Shi H He M Cui L Wang S Zhao J . Associations of caring for grandchildren and great-grandparents with depressive symptoms and life satisfaction in Chinese grandparents: the moderating roles of urban–rural residence and social participation. Fam Process. (2025) 64:e13066. 10.1111/famp.13066 39363509

[67] Choi S-wE Zhang Z. Caring as curing: grandparenting and depressive symptoms in China. Soc Sci Med. (2021) 289:114452. 10.1016/j.socscimed.2021.114452 34624620

[68] Wang S Li S Hu W. Grandparenting and subjective well-being in China: the moderating effects of residential location, gender, age, and income. Soc Sci Med. (2022) 315:115528. 10.1016/j.socscimed.2022.115528 36399982

[69] Liu Y Hughes MC Roberto KA Savla J. Physical and mental health of family caregivers of older parents and grandchildren in China. Aging Health Res. (2022) 2:100052. 10.1016/j.ahr.2021.100052

[70] Wang H Huang J. Impacts of grandparenting on older Chinese adults' mental health: a cross-sectional study. BMC Geriatr. (2023) 23:660. 10.1186/s12877-023-04396-x 37833646 PMC10571259

[71] Hong Y Xu W. Continuity and changes in grandchild care and the risk of depression for Chinese grandparents: new evidence from Charls. Front Public Health. (2023) 11:1217998. 10.3389/fpubh.2023.1217998 37601176 PMC10435994

[72] Wang J Gu R Zhang L Zhang L. How is caring for grandchildren associated with grandparents' health: the mediating effect of internet use. Front Public Health. (2023) 11:1196234. 10.3389/fpubh.2023.1196234 37621608 PMC10446841

[73] Hong Y Xu W Zhao L. The impact of grandchild care on depressive symptoms of grandparents in China: the mediating effects of generational support from children. Front Public Health. (2023) 11:1043969. 10.3389/fpubh.2023.1043969 37020818 PMC10067760

[74] Zeng Y Chen Y-C Lum TYS. Longitudinal impacts of grandparent caregiving on cognitive, mental, and physical health in China. Aging Ment Health. (2021) 25:2053–60. 10.1080/13607863.2020.1856779 33291945

[75] Zou X Lin X Jiang Y Su J Qin S Han ZR. The associations between mothers' and grandmothers' depressive symptoms, parenting stress, and relationship with children: an actor–partner interdependence mediation model. Fam Process. (2020) 59:1755–72. 10.1111/famp.12502 31647575

[76] Silverstein M Zuo D. Grandparents caring for grandchildren in rural China: consequences for emotional and cognitive health in later life. Aging Ment Health. (2021) 25:2042–52. 10.1080/13607863.2020.1852175 33251822

[77] Hsu WC Huang NC Li DC Hu SC. The long-term effects of dual caregiving on the caregivers' well-being among middle-aged and older adults in Taiwan. Aging Ment Health. (2023) 27:1190–7. 10.1080/13607863.2022.2076205 35585725

[78] Tsai F-J. The Maintaining and improving effect of grandchild care provision on elders' mental health—evidence from longitudinal study in Taiwan. Arch Gerontol Geriatr. (2016) 64:59–65. 10.1016/j.archger.2016.01.009 26952378

[79] Kim J Park E-C Choi Y Lee H Lee SG. The impact of intensive grandchild care on depressive symptoms among older Koreans. Int J Geriatr Psychiatry. (2017) 32:1381–91. 10.1002/gps.4625 27905151

[80] Chung S Park A. The longitudinal effects of grandchild care on depressive symptoms and physical health of grandmothers in South Korea: a latent growth approach. Aging Ment Health. (2018) 22:1556–63. 10.1080/13607863.2017.1376312 28910153

[81] Choi J Jun HJ Kim HK. Supplementary grandchild care, social integration, and depressive symptoms: longitudinal findings from Korea. Aging Ment Health. (2021) 25:78–85. 10.1080/13607863.2019.1673307 31591907

[82] Tang F Xu L Chi I Dong X. Psychological well-being of older Chinese-American grandparents caring for grandchildren. J Am Geriatr Soc. (2016) 64:2356–61. 10.1111/jgs.14455 27641829 PMC5118144

[83] Provenzano AM Stearns MA Nadorff DK. The influence of caregiving on the relation between marital status and psychological health in a grandparent sample. Int J Aging Human Dev. (2021) 92:411–30. 10.1177/0091415020920000 32378416

[84] Yalcin BM Pirdal H Karakoc EV Sahin EM Ozturk O Unal M. General health perception, depression and quality of life in geriatric grandmothers providing care for grandchildren. Arch Gerontol Geriatr. (2018) 79:108–15. 10.1016/j.archger.2018.08.009 30196143

[85] Danielsbacka M Tanskanen AO Coall DA Jokela M. Grandparental childcare, health and well-being in Europe: a within-individual investigation of longitudinal data. Soc Sci Med. (2019) 230:194–203. 10.1016/j.socscimed.2019.03.031 31030010

[86] Di Gessa G Glaser K Tinker A. The health impact of intensive and nonintensive grandchild care in Europe: new evidence from share. J Gerontol. (2015) 71:867–79. 10.1093/geronb/gbv055 26315046 PMC4982385

[87] Arpino B Bellani D. Juggling grandchild care and labor force participation: the effect on psychological wellbeing of older women. Front Sociol. (2021) 6:806099. 10.3389/fsoc.2021.806099 35127889 PMC8807505

[88] Ratnakaran B Shappell AV Khalid K. Grandparenting and the golden years: understanding the factors and mental health outcomes of grandparent caregivers in older adults. Am J Geriatr. Psychiatry. (2024) 32:S10. 10.1016/j.jagp.2024.01.053

[89] Ge T Jin S. Social engagement and geriatric depression: under the pension system and the economic environment in China. Curr Psychol. (2023) 42:10871–9. 10.1007/s12144-021-02380-5

[90] Felix MS. Scoping review: obese elderly women with breast cancer and physical activity/exercise. Glob Health J. (2022) 6:129–35. 10.1016/j.glohj.2022.07.011

