# Supplementary material for: The impact of grandchild caregiving on depression among grandparents: a scoping review
Source: Front Public Health. 2025 Oct 31;13:1696678. doi: 10.3389/fpubh.2025.1696678 (PMC12615245; doi:10.3389/fpubh.2025.1696678)
Supplement: Supplementary file 1 [file Table_1.docx]

**Appendix Table S1 Mapping, analysis and synthesis**

| **No.** | **Reference** | **Focus of the research** | **Methodology** | **Characteristics of participants** | **Geographic location of the study** | **Caregiving Context** | **Measurement of Depression** | **​Main findings of the research** | **Potential gap identified** |
| --- | --- | --- | --- | --- | --- | --- | --- | --- | --- |
| 1 | [1] | To explore how various dimensions of caregiving impact the psychological well-being of older Chinese Americans, highlight the role that childcare plays in their daily lives, and examine the potential challenges involved. The study also aims to inform healthcare professionals on ways to enhance the benefits and reduce the negative outcomes of caregiving through targeted interventions. | Method:  Cross-sectional quantitative  Sampling:  Purposive and convenience sampling.  Data collection:  Face-to-face interviews in participants’ preferred language  Data analysis:  Bivariate analyses, including independent t-tests, Wilcoxon signed-rank tests, chi-square tests, and a continuous version of a negative binominal regression model. SAS version 9.2. | Of 2,365 respondents who is Chinese Americans aged 60 and older, 35% (n = 818) were designated as caregivers. The average age of the participants was 72.8, and 42% were male. | The Greater Chicago Area, U.S. | (1) Caregiving or not, (2) caregiving hours (times spent caring for grandchildren per week), (3) self-reported caregiving stress, (4) self-reported caregiving burden (ranging from 0 (never) to 4 (always)) | Patient Health Questionnaire-9 (PHQ-9) | Non-caregivers showed higher levels of depression, anxiety, stress, and loneliness than caregivers. While caregiving time was not linked to mental well-being, perceived burden, pressure, and negative impact significantly raised depression and stress levels. | Grandparents under the age of 60 could be studied. Differences in caregiving roles (occasional caregiver, co-residential caregiver, primary caregiver) and the length of caregiving experience on grandparent depression could also be studied, with differences in this effect between grandfathers and grandmothers. |
| 2 | [2] | To examine the link between providing intensive care for grandchildren and the presence of depressive symptoms in older adults with at least one grandchild. | Method:  Longitudinal quantitative research.  Sampling:  A nationally representative survey which is stratified sampling based on area and housing type.  Data collection:  Data were obtained from the 2008, 2010, and 2012 waves of the Korea Longitudinal Study of Aging (KLoSA).  Data analysis:  Variance, t-tests, and generalized estimating equation analysis. SAS version 9.4. | In the baseline year 2008, old adults without depression who had at least one grandchild and were older than 50 years old (n=5129) were included. 95.1% of grandparents did not provide grandchild care, and 40.7% of respondents were male. | Nationwide, South Korea | How much time is spent caring for them per week ((1) no grandchild care (0 h); (2) non-intensive grandchild care (1–39 h); and (3) intensive grandchild care (more than 40 h). ) | The 10-item version of the CES-D scale. | Intensive grandchild caregiving was linked to reduced depression among older adults. This effect was not observed in non-intensive caregivers or non-caregivers, and was more pronounced in grandfathers than grandmothers. | Grandparents under 50 years of age may still need to be studied, even if this group is small. Characteristics of cared-for grandchildren, especially the effect of age, on caregiver depression. |
| 3 | [3] | To explore how parenting stress mediates the link between depressive symptoms in mothers and grandmothers and their interactions with children within dyadic relationships, and to assess differences across generations. | Method:  Cross sectional research, quantitative.  Sampling:  Purposive and convenience sampling.  Data collection:  Students from the selected families would take home questionnaires. Mothers and grandmothers were required to complete the questionnaires.  Data analysis:  Full-information maximum likelihood (FIML) method was used to account for missing data in Mplus 7.11. APIMeM model was employed to examine the relationship between depressive symptoms and parenting stress. | The sample consisted of 136 pairs of mothers and grandmothers, all from households where grandmothers had provided grandchild care during the previous year and lived in intact families with both parents and a grandmother present. Among the grandmothers involved, 52.94% were from the paternal side and 41.18% from the maternal side. The mean age of grandmothers in the study was approximately 64 years. The grandchildren being cared for ranged in age from 7 to 12 years. | Beijing, China | The Parenting Stress Index Short Form (PSI-SF) was used to measure the parenting stress of grandmother. | The full 20-item version of the CES-D scale | Depression among grandmothers was closely tied to parenting stress and indirectly linked to grandmother–child conflict through both their own and the mother’s stress levels. | Parenting stress and depression among grandfathers, as well as among grandmothers caring for grandchildren under seven years old. Differences in the relationship between caregiving stress and depression among maternal and paternal grandparents. |
| 4 | [4] | To analyze how caregiving for grandchildren and/or great-grandparents is related to depressive symptoms and life satisfaction in Chinese grandparents, and to examine whether these associations are influenced by urban-rural context and levels of social engagement. | Method:  Cross sectional research, quantitative.  Sampling:  Nationally representative sampling.  Data collection:  Data were derived from the China Health and Retirement Longitudinal Study (CHARLS) 2018.  Data analysis:  Harman single-factor test, unrotated factor analysis, chi-square test, Wilcoxon two-sample test, binary logistic regression model was used to analysis the data. SPSS software version 24. | 2973 participants who were 45-75 years old and had at least one parent or parent-in-law and their spouse's parent or parent-in-law alive were included. All participants had at least one grandchild. 48.0% of them were male. | Nationwide, China | The care provision were integrated as neither, grandchild(ren) only, parent(s) only, nor both. | The 10-item version of the CES-D scale. | No significant association was found between caregiving and depression overall. However, in urban areas, caring for great-grandchildren or both grandchildren and great-grandparents was linked to higher depression risk. | The impact of additional caregiving characteristics—such as caregiving roles, length of caregiving experience, and caregiving intensity—on grandparents’ depression. |
| 5 | [5] | To assess the impact of a new form of informal caregiving—directed toward older adults, grandchildren, or both—on the caregivers’ psychological well-being. | Method:  A time-lagged design, quantitative.  Sampling:  nationally representative longitudinal survey.  Data collection:  Data were selected from the Taiwan Longitudinal Study on Aging (TLSA) database, 4 waves from 1996 to 2007..  Data analysis:  Descriptive statistics with mean, standard deviation, frequency, and percentage. The Generalized Estimation Equation (GEE) model. | The baseline sample included 5131 respondents aged over 50. All respondents were non-institutionalized and reported no difficulty with any activities of daily living. 55.2% of the respondents were male. | Taiwan | Grandchildren caregiving was defined as the frequency with which grandparents assisted or babysat their grandchildren on behalf of their adult children. Grandchildren were identified as a child younger than 16 years old. | The 10-item Chinese version of CDS-D Scale. | Older caregivers showed lower life satisfaction and higher depression. Dual caregivers reported greater life satisfaction but more depression than non-caregivers, while those caring only for grandchildren showed no significant differences. | The effect of length of care on grandparent depression. Social support, especially family support, may change the long-term impact of grandchild care. |
| 6 | [6] | To determine whether involvement in grandparenting is linked to depressive symptoms in older adults, and to evaluate whether this relationship is moderated by financial transfers and emotional support from adult children. | Method:  Cross-sectional quantitative research  Sampling:  Purposive and convenience sampling.  Data collection:  Face-to-face interviews.  Data analysis:  Multiple regression models | 99 self-identified Mexican Americans who were 60 years of age or older and had at least one living child were included in the study. 41% of the participants were male. | Lubbock in Texas, U.S. | The measure reflects the total number of weeks a grandparent provided care for any grandchild, distinguishing between non-residential occasional care and full-time residential caregiving. | The full 20-item version of the CES-D scale | Greater caregiving intensity was linked to higher depression levels, with financial support to adult children moderating this effect. Lower support was related to less depression, whereas higher support was linked to greater depression. | Other forms of intergenerational support (such as instrumental or informational support) may also influence the association between grandparental caregiving and depression. |
| 7 | [7] | To explore the connection between grandparental involvement and depression among Chinese older adults. | Method:  Longitudinal quantitative research  Sampling:  Nationally representative sampling.  Data collection:  The study used three waves of CHARLS from 2011 to 2015.  Data analysis:  fixed effects regression models, multiple imputation. Stata 16. | At baseline, 4354 participants who were 45-80 year old and had grandchildren were included. | Nationwide, China | Grandchild caregiving was classified into five types based on caregiving hours and family arrangement: (1) no caregiving role, (2) caregiving in skipped-generation households, (3) caregiving in multigenerational households, (4) part-time caregiving without co-residence, and (5) full-time caregiving without co-residence. | The 10-item version of the CES-D scale. | Skipped-generation caregiving (grandparents raising grandchildren without parents) was associated with lower depression. No major differences were found among other caregiving types. | Differences in the effects of grandchild caregiving on depression in grandfathers and grandmothers. The effects of grandchild caregiving vary depending on the characteristics of the care recipient (i.e., grandchild) and the caregiving support available. |
| 8 | [8] | To assess depression, perceived quality of life, and general health status among caregiving grandmothers, along with the influencing factors. | Method:  Longitudinal quantitative research.  Sampling:  Purposive and convenience sampling.  Data collection:  General questionnaire, the beck depression inventory (BDI), SF-12 v 2 (self-function 12 version 2), EQ-5D-5 visual analog scale were used to collect data.  Data analysis:  the Chi-square, independent samples t-tests and binary logistic regression models. SPSS version 15 and Minitab version 15. | 2563 elderly (older than 65) female respondents were included. | Samsun, Amasya, Canakkale, and Izmir in Turkey | Caregiving was divided into two groups, (1) subjects reporting personally caring for at least one grandchild for at least 50 h in the previous three months, (2) control group. The study group was further divided into three subgroups (babysitters, co-residential, and custodial) based on the types of care provided | The 21-item beck depression inventory (BDI). | Around 11% of older grandmothers were caregivers. Except for custodial caregivers, those who cared for grandchildren had better physical and mental health, lower depression, and higher life quality. | The relationship between grandchild caregiving and depression among the grandmother or grandfather who was middle aged. |
| 9 | [9] | To explore the bidirectional relationship between variations in grandchild care frequency and shifts in grandparents’ health and well-being over time. | Method:  Longitudinal quantitative research.  Sampling:  Nationally representative sampling  Data collection:  Five waves of the SHARE data were used.  Data analysis:  Multilevel Poisson regression, between- and within-person (or fixed-effect) models | A total of 41,713 individuals from 24,787 populations aged 50-89 years were included in the study. 39.9% of the participants were male. | Eleven European countries include Denmark, Sweden, Austria, France, Germany, Switzerland, Belgium the Netherlands, Spain, Italy and Greece. | (1) Grandchild care provision. (2) The score of grandchildren caregiving frequency. | The 12-item European Depression Scale (EURO-D). | In Europe, caregiving grandparents reported better health, less depression, and fewer ADL difficulties, although these links were mostly explained by individual differences. | The impact of grandchild caregiving on different dimensions of depression, such as somatic, emotional, and cognitive symptoms. |
| 10 | [10] | To analyze how grandparenting relates to subjective well-being in older Chinese adults and whether this relationship differs by gender, location, age, and income levels. | Method:  Longitudinal quantitative research.  Sampling:  Nationally representative sampling  Data collection:  The data in this study come from three waves (2011, 2013, and 2015) of the CHARLS.  Data analysis:  pooled ordinary least squares (POLS) regression. | At baseline, a total of 2870 samples were included in the study, with an age range of 50-80. 50% of them were male. | Nationwide, China | Based on the average number of hours per week spent caring for grandchildren under age 16, caregiving levels were classified as follows: no involvement (reference group), low (under 24 hours), moderate (24 to 44 hours), and high (above 44 hours). | The 10-item version of the CES-D scale. | No significant relationship was found between grandparental caregiving and depression. | The influence of different types of caregiving activities and grandchildren's characteristics on the association between grandchildren caregiving and depression. |
| 11 | [11] | To evaluate the effects of caring for younger generations on grandparents’ physical and psychological health. | Method:  Cross-sectional quantitative research  Sampling:  Nationally representative sampling.  Data collection:  The data were obtained from the CHARLS 2018.  Data analysis:  OLS model, propensity score matching (PSM) and instrumental variable model were used to analysis with Stata version 16. | 7821 respondents aged above 45 were included. 52% of them were male. | Nationwide, China | (1) Care involvement was determined by whether the individual had provided any grandchild care in the previous year. (2) Care intensity was assessed through the respondent’s reported average hours of care provided per week. | The 10-item version of the CES-D scale. | Providing care was linked to better physical health and lower depression scores in grandparents, particularly among women, those aged 45–60, and rural residents. These benefits were mainly driven by enhanced economic help, emotional support, and social engagement. |  |
| 12 | [12] | To investigate how fluctuations in older adults’ caregiving for grandchildren correspond to shifts in their mental health. | Method:  Longitudinal quantitative research  Sampling:  Nationally representative sampling  Data collection:  The information from the fifth and sixth waves of the Study of Health and Living Status of the Middle-Aged and Elderly which were conducted in 2003 and 2007.  Data analysis:  ANOVA test, paired-t test, chi-square test and multiple logistic regression were used to analysis with SPSS version 18.0. | 2930 elders aged 50 years and over who had at least one grandchild were included. 45.39% of them were male. | Taiwan | Changes in grandchild care status: always caring for grandchildren, only caring for grandchildren in the first wave, only caring for grandchildren in the second wave, never caring for grandchildren | The 10-item version of the CES-D scale. | Older adults who stopped caregiving after previously providing care faced a higher risk of depression. | The impact of characteristics such as age of the grandchild being cared for needs to be considered. |
| 13 | [13] | To study the health outcomes of Chinese grandparents involved in dual caregiving roles for both grandchildren and their own elderly parents or in-laws. | Method:  Longitudinal quantitative research.  Sampling:  Nationally representative sampling  Data collection:  The data in this study were from the CHARLS waves 2011 and 2013.  Data analysis:  Ordinary least squares (OLS) models, multinomial logit model, lagged dependent variable models. | The baseline sample included 4645 individuals who had at least one living parent or parent-in-law and at least one grandchild younger than 16 at the time of the survey. Of these respondents, 48.05% were male. | Nationwide, China | Care responsibilities were grouped into four types: caregiving solely for grandchildren under age 16, caregiving solely for great-grandparents (including in-laws), caregiving for both groups simultaneously, and no caregiving provided to either grandchildren or great-grandparents. | The 10-item version of the CES-D scale. | Grandparents solely caring for grandchildren reported better health than non-caregivers. Those caring for both older and younger generations (“sandwich” caregivers) had fewer depressive symptoms. Benefits were greatest for urban grandfathers, while rural grandmothers appeared most at risk. | More detailed information on the impact of grandchild caregiving characteristics on grandparents’ mental health, such as intensive care versus non-intensive care, and differences in the amount of energy grandparents put into caregiving activities. |
| 14 | [14] | To profile family caregivers in mainland China and explore links between their demographics, caregiving roles, and long-term health outcomes. | Method:  Longitudinal quantitative research.  Sampling:  Nationally representative sampling  Data collection:  The data in this study were from the CHARLS waves 2013 and 2018.  Data analysis:  t test, chi-square test, linear regression, negative binomial regression were used to analysis with Stata version 14. | 11203 respondents aged 45 years or above were included. 50.26% of participants were male. | Nationwide, China | (1) Caregiving status was classified as providing care only for parents, only for grandchildren, for both parents and grandchildren, or providing no care. (2) Time commitment was measured by responses to a question asking how many weeks per year and hours per week participants spent caring for parents, grandchildren, or parents-in-law over the previous year. | The 10-item version of the CES-D scale. | Those caring only for grandchildren had higher life satisfaction, better physical function, and fewer depression symptoms. Similar positive mental health outcomes were seen in caregivers looking after only parents. | The combined effects of secondary stressors such as work stressors, relationship stressors, and balancing child care on grandparents' mental health. |
| 15 | [15] | To assess the connection between grandparenting and depressive symptoms among older Chinese adults, and to evaluate how different types of intergenerational support affect this relationship. | Method:  cross-sectional quantitative research.  Sampling:  Nationally representative sampling  Data collection:  The data was from the China Longitudinal Aging Social Survey (CLASS) 2018 wave.  Data analysis:  ordinary least squares (OLS) method was used in this study with Stata 17.0. | 4,128 respondents were included in the study. They should have at least one grandchild. 44.6% of them were male. | Nationwide, China | (1) Grandchild care provision. (2) average grandchild care intensity. (3)care intensity level: intensive grandparenting, non-intensive grandparenting, no care. | The 9-item version of the CES-D scale. | Non-intensive grandparenting was associated with lower levels of depression in older adults. Support from adult children acted as a mediator in this relationship. | The effect of other characteristics of care provision on mental health, such as Care responsibility, care content, care stress, coping strategies, and demographic attributes and health conditions of grandchildren. |
| 16 | [16] | To investigate how grandparenting influences depression in Chinese older adults, considering the mediating role of intergenerational assistance. | Method:  longitudinal quantitative research.  Sampling:  Nationally representative sampling  Data collection:  The data was from the CLASS 2014 and 2018 waves.  Data analysis:  ANOVA, chi-square test, the Pooled Ordinary Least Square method (POLS). | A total of 9386 elderly people aged 60-80 years without functional impairment who had at least one grandchild were included in the study. Of these, 45.68% were male. | Nationwide, China | Grandchild care intensity (self-report): non-caregivers, non-intensive caregiving, intensive caregiving. | The 9-item version of the CES-D scale. | Intensive caregiving was linked to improved mental health among grandparents. This relationship was influenced by the financial and practical support provided to adult children. | The associations between grandparenting and depression may differ among different subgroups, including urban and rural, male and female grandparents. |
| 17 | [17] | To explore how changes in grandchild caregiving during the early months of the COVID-19 pandemic relate to grandparents' mental health outcomes. | Method:  cross-sectional quantitative research.  Sampling:  Nationally representative sampling  Data collection:  Using data from the nationally representative English Longitudinal Study of Ageing (ELSA).  Data analysis:  logistic and linear models, Stata 16. | 2468 grandparents aged 50 years or older with at least one grandchild under the age of 15 were recruited. 43.6% were male. | Nationwide, England | Changing status of grandchild care during the pandemic: No grandchild care pracademic, mostly same or increased, mostly decreased or interrupted, completely stopped. | The 8-item version of the CES-D scale. | Grandparents who stopped or reduced caregiving during the pandemic experienced worse mental health than those who continued or increased their care involvement. | The impact of detailed information of childcare provision on grandparents’ mental health. |
| 18 | [18] | To examine how variations in caregiving roles over time affect grandparents’ depressive symptoms, while accounting for subgroup disparities. | Method:  Longitudinal quantitative research.  Sampling:  Nationally representative sampling  Data collection:  The data in this study were from the CHARLS waves 2015 and 2018.  Data analysis:  Ordinary least squares (OLS) models, multinomial logit model, lagged dependent variable models. | At baseline, 9138 respondents aged 45 years and over who had at least one grandchild were included. 48.14% of them were male. | Nationwide, China | (1) The level of grandchild caregiving was categorized into high-intensity (40 or more hours per week or over 2080 hours annually), low-intensity (fewer than 40 hours weekly or under 2080 hours per year), and no caregiving. (2) Caregiving changes were assessed by comparing grandchild care status across two survey waves. | The 10-item version of the CES-D scale. | Reduced or low-level caregiving was linked to less depression compared to no care. This mental health benefit was especially evident among urban grandmothers providing ongoing support. | The specific content of grandparents' activities, characteristics of their grandchildren, shared caregiving situations, and reasons for changes in caregiving practices all influence grandparents' depression. |
| 19 | [19] | To investigate the long-term link between depressive symptoms and caregiving intensity, and explore whether this varies by gender, living arrangements, and location among Chinese grandparents. | Method:  Longitudinal quantitative research.  Sampling:  Nationally representative sampling  Data collection:  The data in this study were from the CHARLS waves 2011, 2013 and 2015.  Data analysis:  ANOVA and chi-square test, multilevel mixed-effects linear regression models were used with Stata 16. | Participants were 4424 respondents aged 50 years and above with at least one grandchild under 16 years of age, of whom 52.30% were male. | Nationwide, China | Grandparental caregiving engagement was classified by time commitment into four groups: high (more than 40 hours per week or 2,080 hours annually), moderate (10–40 weekly hours or 520–2,080 hours annually), low (under 10 weekly hours or a total below 520 hours per year), and non-caregivers (no hours provided). | The 10-item version of the CES-D scale. | Providing moderate levels of care was tied to lower initial depression levels. Low-level caregivers living with a partner or other family members also reported fewer depressive symptoms than non-caregivers in similar living settings. | Information on caregiving responsibilities and context, such as burden or stress, and grandparent-grandchild relationships, may influence grandparent depression. |
| 20 | [20] | To explore gender-based differences in depressive symptoms among rural Chinese grandparents involved in caregiving, informed by role strain and role enhancement theories. | Method:  Cross sectional research, quantitative.  Sampling:  Nationally representative sampling.  Data collection:  Data were derived from the CHARLS 2015.  Data analysis:  Chi-square and ANOVA test, multiple linear regression models, the Coarsened exact matching (CEM) method were used with Stata 15. | A total of 4833 grandparents with "Agricultural Hukou" under the household registration system and with at least one grandchild under 16 years of age were included. The sex ratio of the sample was not reported. | Nationwide, China | (1) Whether the participant provided care for grandchildren. (2) Weekly caregiving hours recorded as a continuous variable. (3) Intensity of care categorized as low (1–14 hours/week), moderate (15–39 hours/week), and high (40 or more hours/week). | The 10-item version of the CES-D scale. | Grandmothers tended to engage in more intensive and longer caregiving than grandfathers. They also reported higher depression levels, with the gender gap widening under high-intensity caregiving conditions. | The relationship between grandchild care provided by grandparents and depression in an urban area, and the influence of grandchild details (e.g., age, gender, health status). |
| 21 | [21] | This research explores gender differences in mental health outcomes between grandmothers and grandfathers involved in caregiving, and the potential moderating role of social participation. | Method:  Longitudinal quantitative research.  Sampling:  Nationally representative sampling  Data collection:  The data come from the Health and Retirement Study (HRS) waves 2002 to 2012.  Data analysis:  ANOVA and chi-square test, multilevel mixed-effects linear regression models were used with Stata 16. | At baseline, 18,383 grandparents who reported having at least one grandchild were included. 39.83% were male. | Nationwide, U.S. | The measure of grandchild caregiving was based on hours of care provided and household composition over the past two years, categorized into five groups: (1) 100–500 hours of care without co-residing grandchildren, (2) more than 500 hours of care without co-residing grandchildren, (3) living with both at least one child and one grandchild (multigenerational setting), (4) living only with grandchildren in the absence of adult children (skipped-generation arrangement), and (5) no caregiving provided. | The 8-item version of the CES-D scale. | Providing care without co-residence enhanced mental health. Grandfathers in skipped-generation households showed poorer mental well-being, while social engagement appeared to mitigate negative outcomes across all groups. | The frequency or continuity of grandchild care may have different effects on grandparent depression. |
| 22 | [22] | This article investigates how caregiving for grandchildren affects emotional and cognitive health in grandparents, and whether financial support from adult children buffers the impact of intensive caregiving. | Method:  Longitudinal research, quantitative.  Sampling:  Random stratified multistage method.  Data collection:  Data derived from six waves of the Longitudinal Study of Older Adults in Anhui Province, China.  Data analysis:  The lagged person-interval regression, Stata v.14.2. | A total of 1432 individuals older than 60 years were included in the study; these individuals had to be grandparents with at least one grandchild under the age of 16 in at least one survey wave. 50.4% were male. | Anhui Province, China | (1) The extent of caregiving was assessed based on how frequently grandparents cared for grandchildren. (2) Household structure was categorized as either three-generational or skipped-generational, depending on co-residence patterns with grandchildren and adult children. | The 9-item version of the CES-D scale. | Frequency of caregiving did not notably affect emotional or cognitive well-being, though negative impacts were observed in custodial households with limited financial support from adult children. | Characteristics of grandchildren, especially behavioral, emotional, and health problems, may contribute to depression in caregivers of grandparents. |
| 23 | [23] | This study explores the relationship between grandparental involvement in childcare and their health outcomes over two- and four-year follow-up periods. | Method:  Longitudinal quantitative research.  Sampling:  Nationally representative sampling  Data collection:  The data was from waves 1–4 of the SHARE.  Data analysis:  Logistic regression model, Stata 13. | 15,374 respondents with at least one grandchild and aged 50 or above were included in the study at baseline, and only grandparents who participated in all four waves were considered in subsequent analyses. 41.6% were male. | Eleven European countries include Denmark, Sweden, Austria, France, Germany, Switzerland, Belgium the Netherlands, Spain, Italy and Greece. | (1) Whether the respondent provided grandchild care. (2) Care intensity was defined by frequency and duration: intensive care referred to caregiving almost every day or at least 15 hours weekly, while non-intensive care included caregiving weekly for less than 15 hours, monthly, or less frequently. | The 12-item European Depression Scale (EURO-D). | Engaging in either intensive or non-intensive grandchild care was associated with better self-rated health, though no clear link to depression was found. | Gender differences in grandchild care may have different impacts on grandparents' health. |
| 24 | [24] | This study focuses on the mediating role of Internet usage in the relationship between caregiving for grandchildren and grandparents’ health outcomes. | Method:  Cross sectional research, quantitative.  Sampling:  Nationally representative sampling.  Data collection:  Data were derived from the CHARLS 2018.  Data analysis:  Chi-square test and t-test, logistic probability model, the KHB method. | 16,829 individuals aged 50-80 years with at least one grandchild were included in the study. 47.89% were male. | Nationwide, China | (1) Whether the respondent provided care for grandchildren. (2) Daily caregiving time was measured by the number of hours the participant or their spouse spent caring for grandchildren each day. | The 10-item version of the CES-D scale. | Caring for grandchildren was linked to improved mental well-being, greater life satisfaction, and lower IADL limitations. Internet use played a mediating role in enhancing these health outcomes. | Studies with longer time spans can lead to more reliable conclusions. |
| 25 | [25] | This research explores the long-term influence of grandchild caregiving on grandparental depression, including the mediating role of support from their adult children. | Method:  Longitudinal quantitative research.  Sampling:  Nationally representative sampling.  Data collection:  The data in this study were from the CHARLS waves 2015 and 2018.  Data analysis:  The Ordinary Least Squares (OLS) model, the KHB model, Stata 17. | 9134 respondents aged 45 and above who reported having at least one grandchild under 16 years old. 49% of the samples were male. | Nationwide, China | Grandchild care provision. | The 10-item version of the CES-D scale. | Caring for grandchildren was significantly associated with reduced depression, particularly when supported by instrumental help, followed by financial and emotional assistance. | It's necessary to explore how additional psychosocial factors, such as stress, sleep quality, or perceived emotional support which may mediate or moderate the relationship between grandchild caregiving and grandparents’ depressive symp-toms. |
| 26 | [26] | This study assesses how marital status affects the link between caregiving and depressive symptoms among grandparents at the national level. | Method:  Cross sectional research, quantitative.  Sampling:  Nationally representative sampling.  Data collection:  Data were come from the third wave of the National Survey of Families and Households (NSFH).  Data analysis:  Correlation and t-tests, linear regression model, hierarchical logistic regression model, SPSS. | Data were drawn from 3288 grandparents who indicated they were either married, divorced, or separated for marital reasons. Among them, 39.7% were male. | Nationwide, U.S. | Primary grandchild care provision | The 12-item version of the CES-D scale. | Depression levels were higher among caregiving grandparents, women, and those without a spouse. However, caregiving did not change the link between marital status and depression. | Social support may mediate the relationship between grandchild care and grandparent depression. |
| 27 | [27] | This research analyzes how combining employment and grandchild caregiving responsibilities influences grandparents’ psychological well-being. | Method:  Longitudinal quantitative research.  Sampling:  Nationally representative sampling.  Data collection:  The data was from the SHARE except for the 3rd wave and 8th wave.  Data analysis:  panel data linear models. | The criteria for sample selection were not reported, but all included samples were female grandmothers. | Eleven European countries include Denmark, Sweden, Austria, France, Germany, Switzerland, Belgium the Netherlands, Spain, Italy and Greece. | Grandchild care provision. | The 12-item European Depression Scale (EURO-D). | Grandmothers engaged in childcare had better quality of life and fewer depressive symptoms than non-caregivers, though life satisfaction remained unaffected. Positive effects were specific to non-working women, while employment was linked to lower depression among those not involved in caregiving. | Differences between co-residential and non-co-residential grandchild caregiving may influence the association between caregiving status and grandparents’ depression. |
| 28 | [28] | This study examines how different caregiving trajectories—continuing, ceasing, or changing care—affect depression and self-perceived health in Korean grandmothers over time. | Method:  Longitudinal quantitative research.  Sampling:  Nationally representative sampling.  Data collection:  Data were come from the KLoSA waves 1-4.  Data analysis:  latent growth curve (LGC) modeling, Mplus. | 1948 female participants who had at least one grandchild and were age 50–74 at baseline were included. | Nationwide, South Korea | Patterns of grandchild care: Never raised grandchildren, transitioned to raising, stopped raising, continuously raised. | The 10-item version of the CES-D scale. | Stopping caregiving was related to higher depression levels among grandmothers, especially those with incomes below 60% of the national median, but not among higher-income individuals. | The influence of family structure, specific caregiving arrangements, and grandchildren’s exact age on the relationship between grandchild caregiving and grandparents’ depression. |
| 29 | [29] | This study analyzed the longitudinal effects of grandparenting on health and considered subgroup differences. | Method:  Longitudinal quantitative research.  Sampling:  Nationally representative sampling  Data collection:  The data in this study were from the CHARLS waves 2011, 2013 and 2015.  Data analysis:  a propensity score method, generalized propensity score analysis, multinomial logistic regression, Stata 14.2. | 4,925 respondents age 45 and older who had grandchildren at baseline were included. 47.63% were male. | Nationwide, China | Grandchild care intensity: No grandparenting care (reference group), Low (those who provided care for at least one grandchild for < 24 h weekly), Moderate (24–44 h weekly), and High (> 44 h weekly) | The 10-item version of the CES-D scale. | Moderate-level caregiving was linked to fewer depression symptoms, whereas low or high intensity care showed no significant effect. Older grandparents benefited more, with no notable differences by gender or region. | The quality and type of grandparent care and characteristics of grandchildren may influence grandparents’ mental health outcomes. |
| 30 | [30] | This study examined the extent to which transitions in grandchild care over time (started, continuous, discontinued, and non-caregiving) were directly and indirectly associated with changes in grandparents’ depressive symptoms through changes in two domains of social integration. | Method:  Longitudinal quantitative research.  Sampling:  Nationally representative sampling.  Data collection:  Data were come from the KLoSA waves 3 and 4.  Data analysis:  bivariate correlation analyses, structural equation modeling, Mplus 8 and SPSS 25. | 4362 respondents aged 45-80 years with at least one grandchild were included, of whom 39.66% were male. | Nationwide, South Korea | Transition patterns of grandchild care status: started caregivers, continuous caregivers, discontinued caregivers, non-caregivers. | The 10-item version of the CES-D scale. | While direct caregiving had no significant link to depression, grandparents who began or continued caregiving experienced improved mental health through enhanced intergenerational interaction. | The effects of caregiving characteristics such as caregiving duration, number of grandchildren, co-residential status, and lineage on grandparents’ depression remain understudied. |

[1] F. Tang, L. Xu, I. Chi, and X. Dong, Psychological Well-Being of Older Chinese-American Grandparents Caring for Grandchildren. Journal of the American Geriatrics Society 64 (2016) 2356-2361.

[2] J. Kim, E.-C. Park, Y. Choi, H. Lee, and S.G. Lee, The impact of intensive grandchild care on depressive symptoms among older Koreans. International Journal of Geriatric Psychiatry 32 (2017) 1381-1391.

[3] X. Zou, X. Lin, Y. Jiang, J. Su, S. Qin, and Z.R. Han, The Associations between Mothers’ and Grandmothers’ Depressive Symptoms, Parenting Stress, and Relationship with Children: An Actor–Partner Interdependence Mediation Model. Family Process 59 (2020) 1755-1772.

[4] H. Zhao, H. Shi, M. He, L. Cui, S. Wang, J. Zhao, W. Li, Y. Wei, W. Zhang, Z. Chen, H. Liu, and X. Zhang, Associations of caring for grandchildren and great-grandparents with depressive symptoms and life satisfaction in Chinese grandparents: The moderating roles of urban–rural residence and social participation. Family Process 64 (2025) e13066.

[5] W.C. Hsu, N.C. Huang, D.C. Li, and S.C. Hu, The long-term effects of dual caregiving on the caregivers’ well-being among middle-aged and older adults in Taiwan. Aging and Mental Health 27 (2023) 1190-1197.

[6] Y. Liu, and Z. Cong, Grandparenting and Depressive Symptoms among Mexican American Older Adults: Examining the Moderating Effects of Financial and Emotional Support. Journal of Intergenerational Relationships 17 (2019) 163-177.

[7] S.-w.E. Choi, and Z. Zhang, Caring as curing: Grandparenting and depressive symptoms in China. Social Science & Medicine 289 (2021) 114452.

[8] B.M. Yalcin, H. Pirdal, E.V. Karakoc, E.M. Sahin, O. Ozturk, and M. Unal, General health perception, depression and quality of life in geriatric grandmothers providing care for grandchildren. Archives of Gerontology and Geriatrics 79 (2018) 108-115.

[9] M. Danielsbacka, A.O. Tanskanen, D.A. Coall, and M. Jokela, Grandparental childcare, health and well-being in Europe: A within-individual investigation of longitudinal data. Social Science & Medicine 230 (2019) 194-203.

[10] S. Wang, S. Li, and W. Hu, Grandparenting and subjective well-being in China: The moderating effects of residential location, gender, age, and income. Social Science & Medicine 315 (2022) 115528.

[11] L. Wang, and Y. Tang, Impacts of intergenerational caregiving on grandparents’ health: Implications for SDG-3. Economic Analysis and Policy 79 (2023) 584-598.

[12] F.-J. Tsai, The maintaining and improving effect of grandchild care provision on elders’ mental health—Evidence from longitudinal study in Taiwan. Archives of Gerontology and Geriatrics 64 (2016) 59-65.

[13] H. Xu, Physical and mental health of Chinese grandparents caring for grandchildren and great-grandparents. Social Science & Medicine 229 (2019) 106-116.

[14] Y. Liu, M.C. Hughes, K.A. Roberto, and J. Savla, Physical and mental health of family caregivers of older parents and grandchildren in China. Aging and Health Research 2 (2022) 100052.

[15] H. Wang, Y. Lu, and J. Huang, All in the Family: Does Grandparenting Impact Chinese Grandparents’ Depressive Symptoms? SAGE Open 14 (2024) 21582440241242561.

[16] H. Wang, and J. Huang, Impacts of grandparenting on older Chinese adults' mental health: a cross-sectional study. BMC geriatrics 23 (2023) 660.

[17] G. Di Gessa, V. Bordone, and B. Arpino, Changes in Grandparental Childcare During the Pandemic and Mental Health: Evidence From England. J Gerontol B Psychol Sci Soc Sci 78 (2023) 319-329.

[18] Y. Hong, and W. Xu, Continuity and changes in grandchild care and the risk of depression for Chinese grandparents: new evidence from CHARLS. Front Public Health 11 (2023) 1217998.

[19] F. Tang, K. Li, H. Jang, and M.B. Rauktis, Depressive symptoms in the context of Chinese grandparents caring for grandchildren. Aging & Mental Health 26 (2022) 1120-1126.

[20] D. Zhao, Z. Zhou, C. Shen, S. Ibrahim, Y. Zhao, D. Cao, and S. Lai, Gender differences in depressive symptoms of rural Chinese grandparents caring for grandchildren. BMC Public Health 21 (2021) 1838.

[21] I.R. Notter, Grandchild Care and Well-Being: Gender Differences in Mental Health Effects of Caregiving Grandparents. J Gerontol B Psychol Sci Soc Sci 77 (2022) 1294-1304.

[22] M. Silverstein, and D. Zuo, Grandparents caring for grandchildren in rural China: consequences for emotional and cognitive health in later life. Aging & Mental Health 25 (2021) 2042-2052.

[23] G. Di Gessa, K. Glaser, and A. Tinker, The Health Impact of Intensive and Nonintensive Grandchild Care in Europe: New Evidence From SHARE. The Journals of Gerontology: Series B 71 (2015) 867-879.

[24] J. Wang, R. Gu, L. Zhang, and L. Zhang, How is caring for grandchildren associated with grandparents' health: the mediating effect of internet use. Front Public Health 11 (2023) 1196234.

[25] Y. Hong, W. Xu, and L. Zhao, The impact of grandchild care on depressive symptoms of grandparents in China: The mediating effects of generational support from children. Front Public Health 11 (2023) 1043969.

[26] A.M. Provenzano, M.A. Stearns, and D.K. Nadorff, The Influence of Caregiving on the Relation Between Marital Status and Psychological Health in a Grandparent Sample. The International Journal of Aging and Human Development 92 (2021) 411-430.

[27] B. Arpino, and D. Bellani, Juggling Grandchild Care and Labor Force Participation: The Effect on Psychological Wellbeing of Older Women. Front Sociol 6 (2021) 806099.

[28] S. Chung, and A. Park, The longitudinal effects of grandchild care on depressive symptoms and physical health of grandmothers in South Korea: a latent growth approach. Aging & Mental Health 22 (2018) 1556-1563.

[29] Y. Zeng, Y.-C. Chen, and T.Y.S. Lum, Longitudinal impacts of grandparent caregiving on cognitive, mental, and physical health in China. Aging & Mental Health 25 (2021) 2053-2060.

[30] J. Choi, H.J. Jun, and H.K. Kim, Supplementary grandchild care, social integration, and depressive symptoms: longitudinal findings from Korea. Aging & Mental Health 25 (2021) 78-85.
